# A Multimodal Approach to a Complex PHACES Patient With Progressive Infantile Hemangioma: A Case Report and Review of Literature

**DOI:** 10.7759/cureus.80261

**Published:** 2025-03-08

**Authors:** Evan D Hicks, Muhammad Hameed, Humam Shahare, Paige Jones-Brooks, Kevin Wong, Jacob Filipek, Gresham T Richter

**Affiliations:** 1 Otolaryngology-Head and Neck Surgery, University of Arkansas for Medical Sciences, Little Rock, USA; 2 Pediatrics, University of Arkansas for Medical Sciences, Little Rock, USA; 3 Pediatric Interventional Radiology, University of South Alabama, Mobile, USA; 4 Pediatrics/Hospital Medicine, University of Arkansas for Medical Sciences, Little Rock, USA

**Keywords:** congenital vascular anomaly, infantile hemangioma, pediatric vascular malformation, phaces, propranolol

## Abstract

A three-week-old baby presented with an infantile hemangioma in segmental beard distribution and evidence of PHACES syndrome (posterior fossa abnormalities, hemangiomas, arterial anomalies, cardiac abnormalities, eye anomalies, and sternal defects). Due to the progressive and symptomatic growth of infantile hemangioma, this patient required a multimodal treatment approach, including beta-blocker therapy, intralesional steroid injections, periorbital surgical debulking, airway interventions, and embolization. This report highlights a successful case of a complex PHACES patient that illustrates the significance of phased multidisciplinary care and emphasizes the importance of compliance. A literature review is included to highlight previously reported successful cases using alternative treatments to propranolol monotherapy. This case offers a unique insight into the timeline and outcomes of a PHACES patient with extensive disease requiring a multimodal approach and highlights possible disease presentation in the non-compliant or resistant patient.

## Introduction

Posterior fossa abnormalities, hemangiomas, arterial anomalies, cardiac abnormalities, eye anomalies, and sternal defects comprise a rare syndrome, PHACES [1-3]. Current literature reviews suggest that there are no more than 400 cases worldwide. However, some suggest the true estimate to be closer to 600 cases [4]. PHACES is diagnosed with the presence of an infantile segmental hemangioma larger than 5 cm on the face, scalp, or cervical region with one major or two minor criteria [5]. Here, we present a patient who met the criteria for PHACES with segmental hemangiomas in a beard distribution and cerebrovascular anomalies, with small right cervical carotid, small right vertebral artery, and fusiform aneurysmal dilation of the left internal carotid artery. Although there is a recent investigation into PHACES, the literature lacks reports on the multimodal treatment of complex PHACES patients with limited response to propranolol monotherapy. We report a unique case of propranolol-resistant PHACES syndrome complicated by hemangiomas on the face, eyelid, larynx, trachea, subglottis, and mediastinum. The patient required intralesional steroid injection, periorbital excision, facial artery collateral embolization, laser therapy, and airway interventions. Although previously thought to be only propranolol-resistant, the patient was also confirmed to be non-compliant in their therapy. This report's objective is to emphasize the need for multimodal therapy in complex PHACES patients in the setting of propranolol resistance and/or non-compliance.

## Case presentation

**Figure 1:**
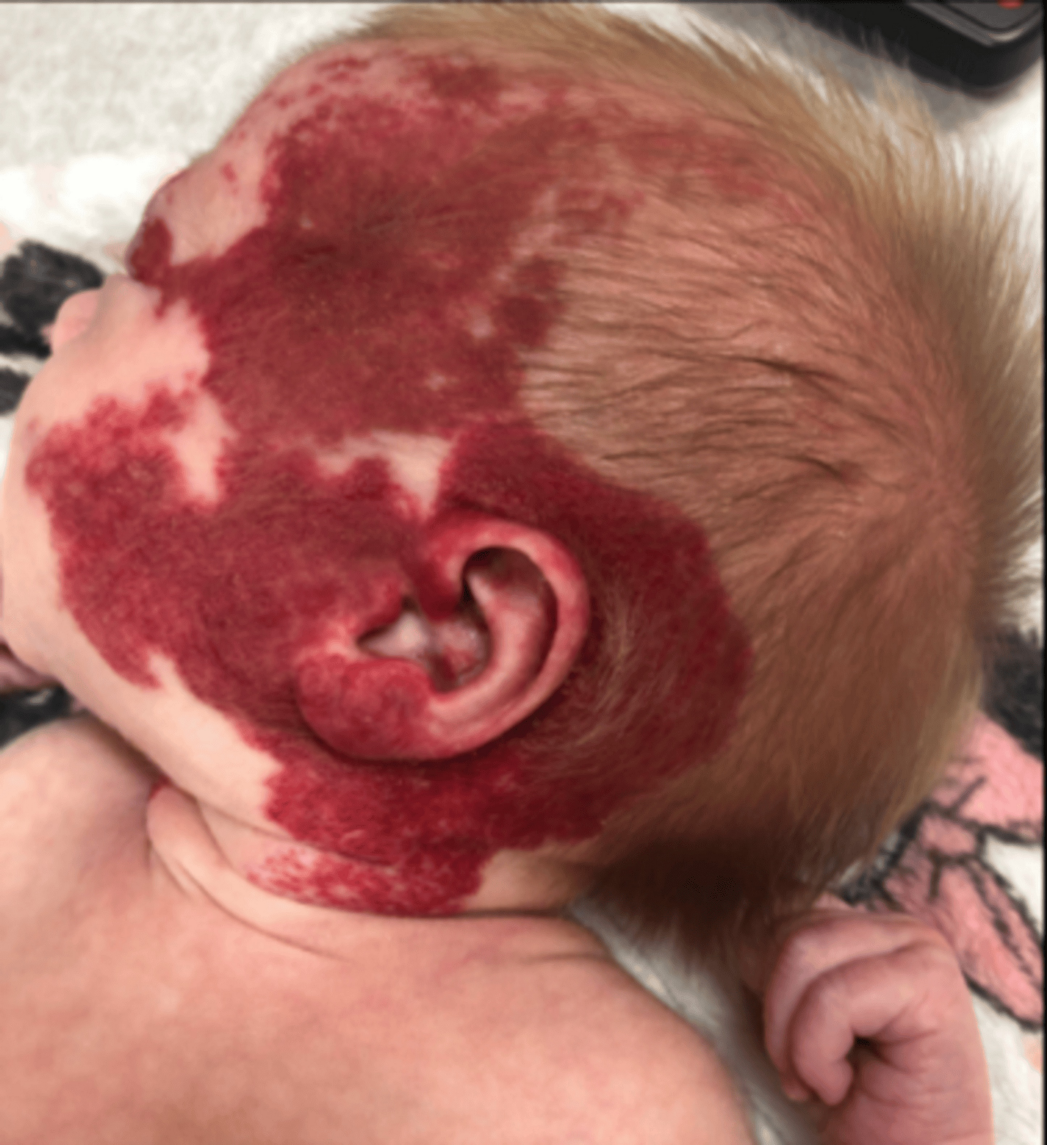
Patient presented with bilateral facial hemangiomas

A three-week-old female baby was presented to the vascular anomaly mini-team clinic with bilateral facial infantile hemangiomas in a beard distribution and stridor (Figure 1). A large hemangioma partially occluded the left eye, and a separate hemangioma partially occluded the left ear canal. Laryngoscopy showed subglottic stenosis, suspected to be a subglottic progressive hemangioma, and mild laryngomalacia.

Ophthalmology

**Figure 2:**
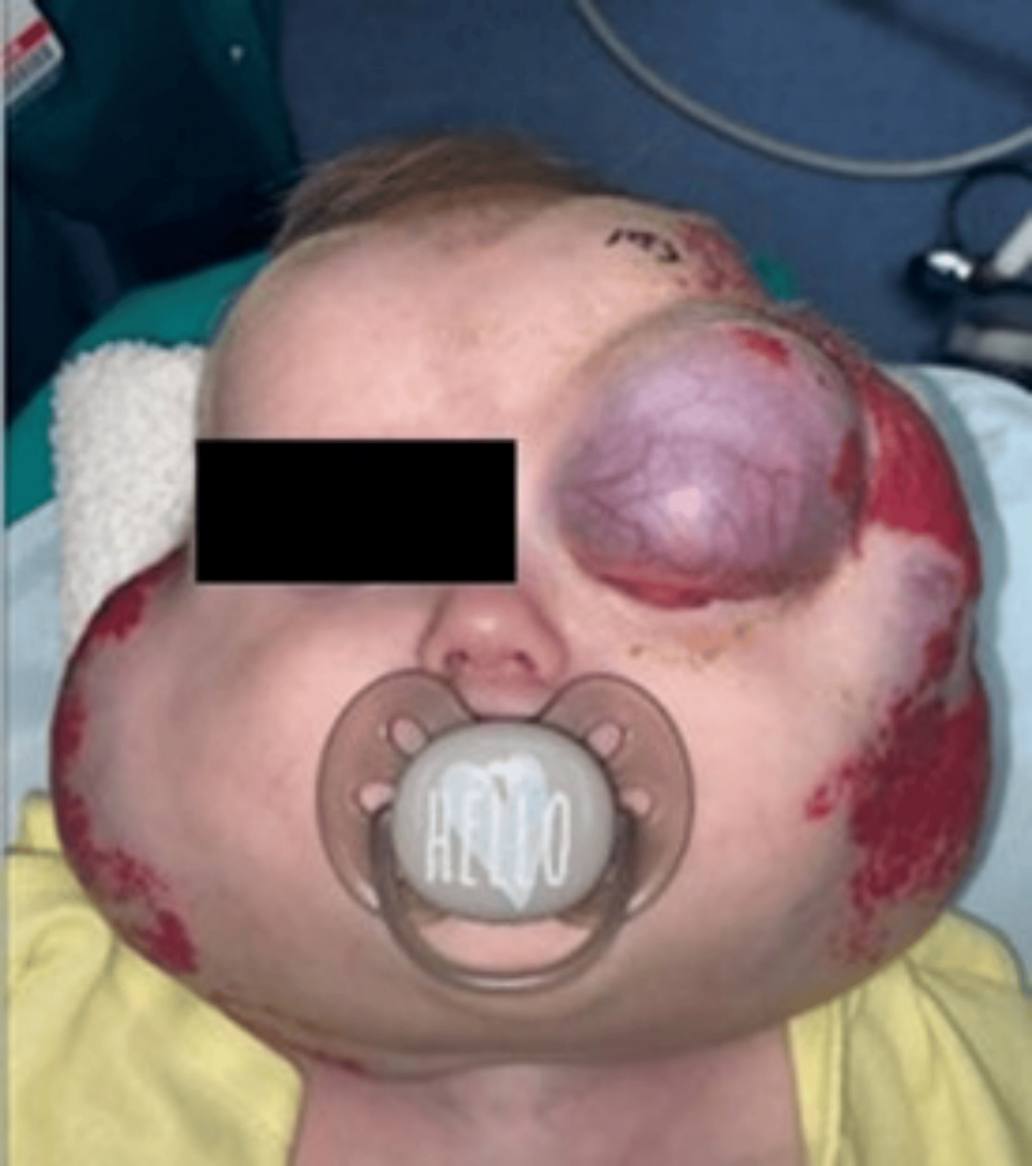
Hemangioma of the left upper eyelid and anterior segment

**Figure 3:**
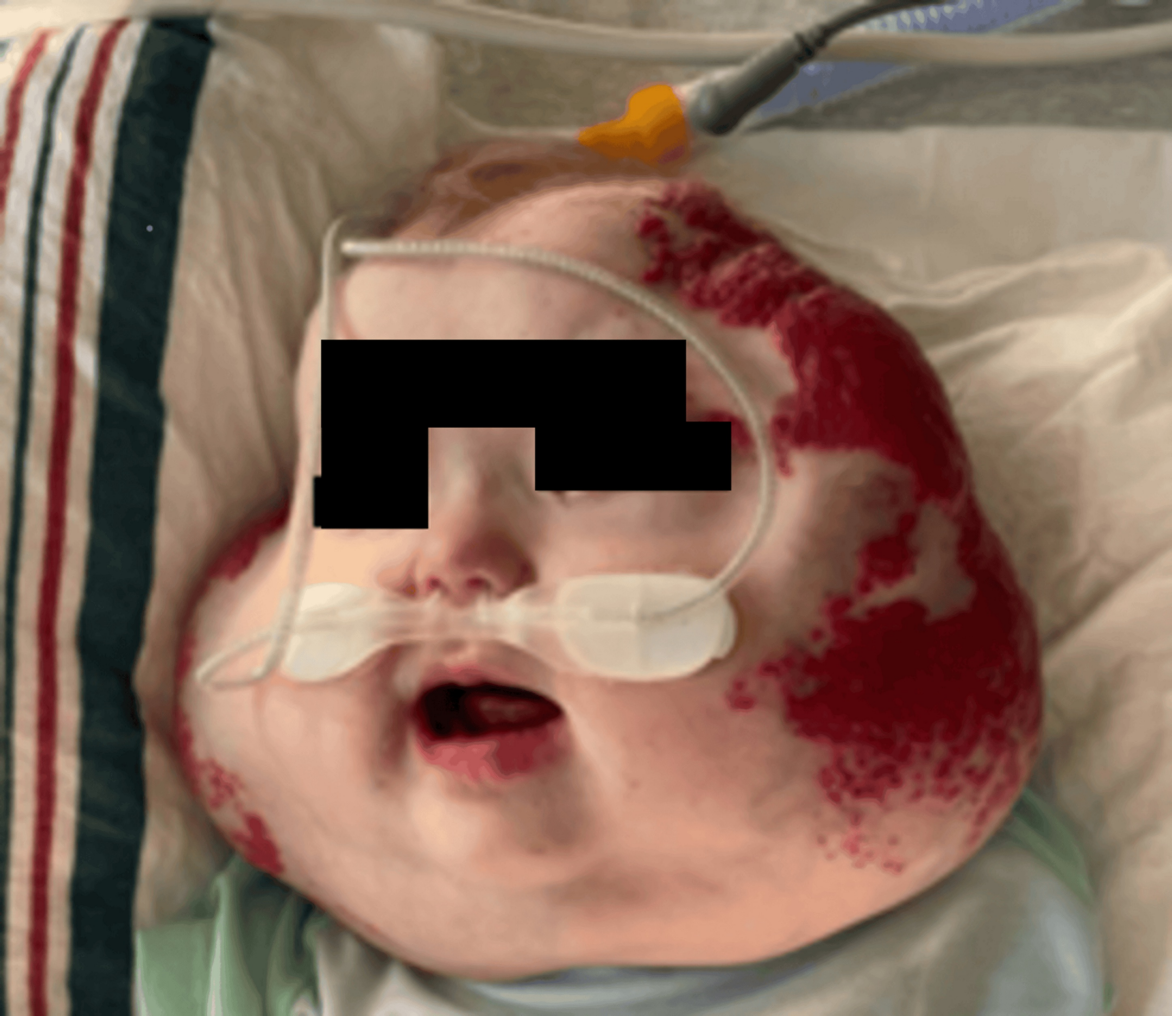
Resolution of the eyelid hemangioma post orbital debulking

Due to social constraints, the patient was lost to follow-up, and five months later, the patient presented with a notable increase in periorbital and facial cutaneous hemangiomas. Periorbital hemangioma presented with visual axis obstruction caused by the eyelid hemangioma (Figure 2). The ophthalmologist performed a debulking procedure for hemangioma of the left upper eyelid and anterior segment, and good clinical outcomes were reported (Figure 3).

Interventional radiology

**Figure 4:**
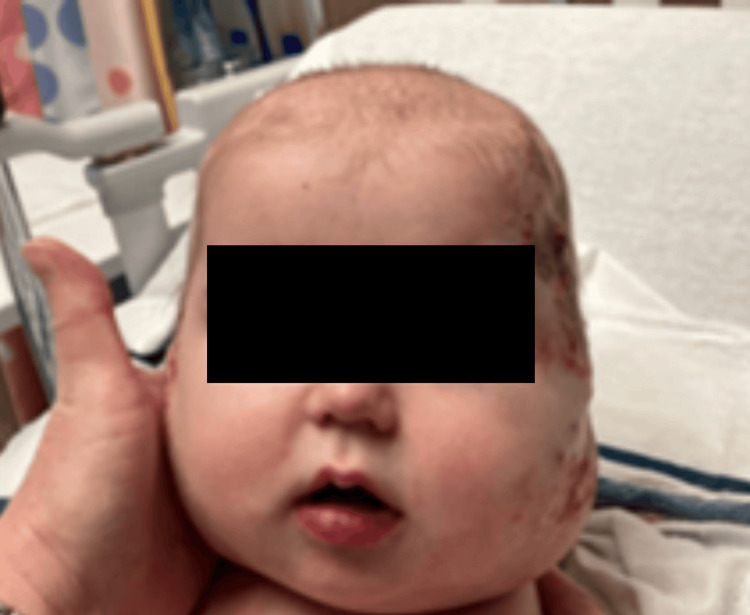
Improvement in facial hemangiomas two months post second IR embolization and propranolol IR: interventional radiology

One month later, she was readmitted for respiratory distress, and larger hemangiomas were noted. Propranolol was started but subsequently switched to atenolol therapy at 0.5 mg/kg due to bradycardia and the hope of better compliance due to once-a-day administration. Atenolol was discontinued due to worsening bradycardia, and propranolol 1.5 mg/kg was restarted with better tolerance. During this time, social work was consulted due to reported non-compliance with home propranolol. Two months later, a microlaryngoscopy and bronchoscopy (MLB) was performed, and a subglottic hemangioma starting posteriorly and extending to the left laterally was visualized. The patient received a third round of triamcinolone injection and proceeded to interventional radiology (IR) for embolization. The left facial artery was identified as the primary blood source of the left facial hemangioma, and embolization was performed on the collateral branches. Three months later, the patient underwent MLB with triamcinolone injections into the left subglottis and parotid glands, with subsequent laser therapy to the cheeks. This was followed by a second round of embolization with IR (Figure 4).

Outpatient care

This patient was under the care of vascular anomalies in the outpatient setting due to an increase in lesion size. A treatment regimen involving a combination of propranolol and steroid therapy was implemented. Hematology/Oncology oversaw anticoagulation using enoxaparin due to the development of cerebral venous thrombosis. Neurology was consulted for the management of seizures and global developmental delay. Seizures were controlled with levetiracetam. Occupational therapy monitored her progress in addressing global developmental delay. Audiology provided a follow-up for hearing loss in her right ear. The patient experienced multiple hospital admissions due to this complex medical course.

## Discussion

The prevalence of infantile hemangiomas is estimated to be about 1%-3% in newborn infants and 2.6%-9.9% in older children [6]. PHACES syndrome is observed in 2%-3% of cases involving infantile hemangiomas [3]. PHACES can present with either multisystem organ involvement or affect a single system. The major and minor diagnostic criteria for PHACES are listed in Table 1 [5]. It is crucial for general pediatricians to understand the multidisciplinary approach and multimodal treatment options for managing complex cases of PHACES syndrome. Additionally, patient education plays a significant role in preventing unnecessary progression of the disease.

**Table 1:** PHACES major and minor diagnostic criteria

Major criteria	Minor criteria
Arterial: anomalies of major cerebral or cervical arteries, stenosis, occlusion, dysplasia, hypoplasia, persistent carotid-vertebrobasilar anastomosis	Arterial: aneurysm of cerebral arteries
Structural brain: posterior fossa anomalies: Dandy-Walker complex, unilateral or bilateral cerebral dysplasia or hypoplasia	Structural brain: midline anomaly, malformation of cortical development, neuronal migration disorder
Cardiovascular: aortic arch anomalies, aneurysm, an aberrant origin of the subclavian artery	Cardiovascular: ventricular septal defect, right aortic arch
Ocular: posterior segment anomaly, retinal vascular anomalies	Ocular: anterior segment anomalies, cataracts, sclerocornea, microphthalmia
Ventral or midline: sternal defect, sternal cleft	Ventral or midline: hypopituitarism, ectopic thyroid, midline sternal papule

Since 2008, propranolol has been the first-line treatment for non-resolving infantile hemangiomas [7,8]. It has been shown to be both safe and effective for treating infantile hemangiomas associated with PHACES syndrome. Propranolol is typically dosed at 1-3 mg/kg/day, divided into two to three doses, with treatment duration ranging from two to 12 months [7]. While most infants tolerate propranolol well, adverse effects such as sleep disturbances, hypotension, and bradycardia have been reported, albeit rarely [8]. In some cases, atenolol has been used as an alternative to propranolol, particularly when bradycardia and hypotension are of concern, although in our case, atenolol worsened bradycardia. Moreover, there is an increased risk of acute ischemic stroke in patients with PHACES due to arterial anomalies, though a recent retrospective study of 76 patients reported no incidence of stroke while on propranolol therapy [7,9].

A literature review was conducted to evaluate reported cases of PHACES patients who either did not respond to propranolol monotherapy or required alternative treatments. Our review highlights that propranolol is the primary accepted treatment for infantile hemangiomas within the context of PHACES syndrome [1]. However, a gap exists in the literature regarding alternative treatments for patients who are either non-responsive to propranolol or have contraindications to its use. Reported alternative medical therapies include combination therapy with steroids and propranolol, as well as other beta-blockers such as topical timolol and systemic atenolol. However, the evidence supporting these alternatives is primarily limited to case reports and a small number of retrospective studies [8,10-19]. Additionally, common procedures reported in these cases include laser therapy, triamcinolone injections, laryngotracheal reconstruction, and surgical debulking [16,17,20-25].

A challenge in treating extensive hemangiomas is the potential for propranolol resistance, the cause of which remains unclear. Treatment interruption and non-adherence have been identified as risk factors for resistance, while genetic factors are being explored [26]. For propranolol-resistant hemangiomas, alternative therapies such as rapamycin, prednisone, and vincristine have been investigated, though no single approach has been universally accepted [27-30].

In this case, the patient did not respond adequately to propranolol, requiring a multispecialty approach due to the extensive nature of the disease. Orbital hemangioma debulking and IR embolization of the facial hemangioma were performed, both of which have been associated with improvements in cosmetic and functional outcomes in propranolol-resistant cases [31-33]. Orbital debulking reduces the risk of ocular sequelae, while embolization decreases the hemangioma's vascularity. For the subglottic hemangioma, interventions such as intralesional steroid injections, laser therapy using microlaser bronchoscopy, and IR embolization were employed to prevent airway complications. Although microlaser bronchoscopy is a non-invasive and effective method for shrinking subglottic hemangiomas, it often requires multiple sessions. Steroid injections have also proven effective for hemangiomas in this location.

Given the lack of standardized guidelines for treating propranolol-resistant hemangiomas, documenting successful cases is essential to establish expectations for PHACES patients requiring a multidisciplinary approach.

The treatment course for propranolol-resistant PHACES syndrome can be lengthy and impose significant emotional and financial burdens on patients and families. In this case, disease progression occurred due to prolonged non-compliance with therapy, emphasizing the importance of patient education and socioeconomic considerations in ensuring proper adherence to treatment. As children with PHACES age, a multidisciplinary approach is often necessary due to the potential for multisystem organ involvement. The general practitioner plays a crucial role in managing these patients by ensuring compliance with medical treatment, facilitating follow-up with specialists, and educating families about the various medical and surgical options available. They serve as the cornerstone of a successful treatment plan through their medical expertise, rapport with patients and families, and consistent follow-up.

## Conclusions

Complex PHACES patients have extensive disease, and this can cause a burden on the family and significant morbidity. In extensive cases, the pediatrician is wise to explore a multimodal approach. It is imperative to understand the course of managing these patients in a multidisciplinary way that involves medical, surgical, and IR management for optimal outcomes. This provides a successful case of a complex PHACES patient treated with beta-blocker therapy, intralesional steroid injections, periorbital surgical debulking, airway interventions, and embolization.

There are limitations due to this being a single case without follow-up data. We have not addressed the long-term effects of the advanced procedures this patient required or the impact an extensive surgical process will have on the family members. Clinicians also need to know how to successfully communicate the required interventions to the parents of patients to decrease hesitancy and promote treatment if required. Clinicians should be encouraged to report more cases of extensive disease requiring multimodal treatment. Future research should be done to investigate successful alternatives to propranolol in efforts to decrease disease severity in those found to be resistant as well as combining modalities in severe cases. Clinicians should also remain aware of factors that contribute toward non-compliance to better address patients with unexpected increasing severity of disease.
